# Supplementary material for: Endogenous indole-3-acetamide levels contribute to the crosstalk between auxin and abscisic acid, and trigger plant stress responses in Arabidopsis
Source: J Exp Bot. 2020 Oct 17;72(2):459–75. doi: 10.1093/jxb/eraa485 (PMC7853601; doi:10.1093/jxb/eraa485)
Supplement: eraa485_suppl_Supplementary_Figures [file eraa485_suppl_supplementary_figures.pdf]

Supplementary Figures S1-S6

Figure S1.

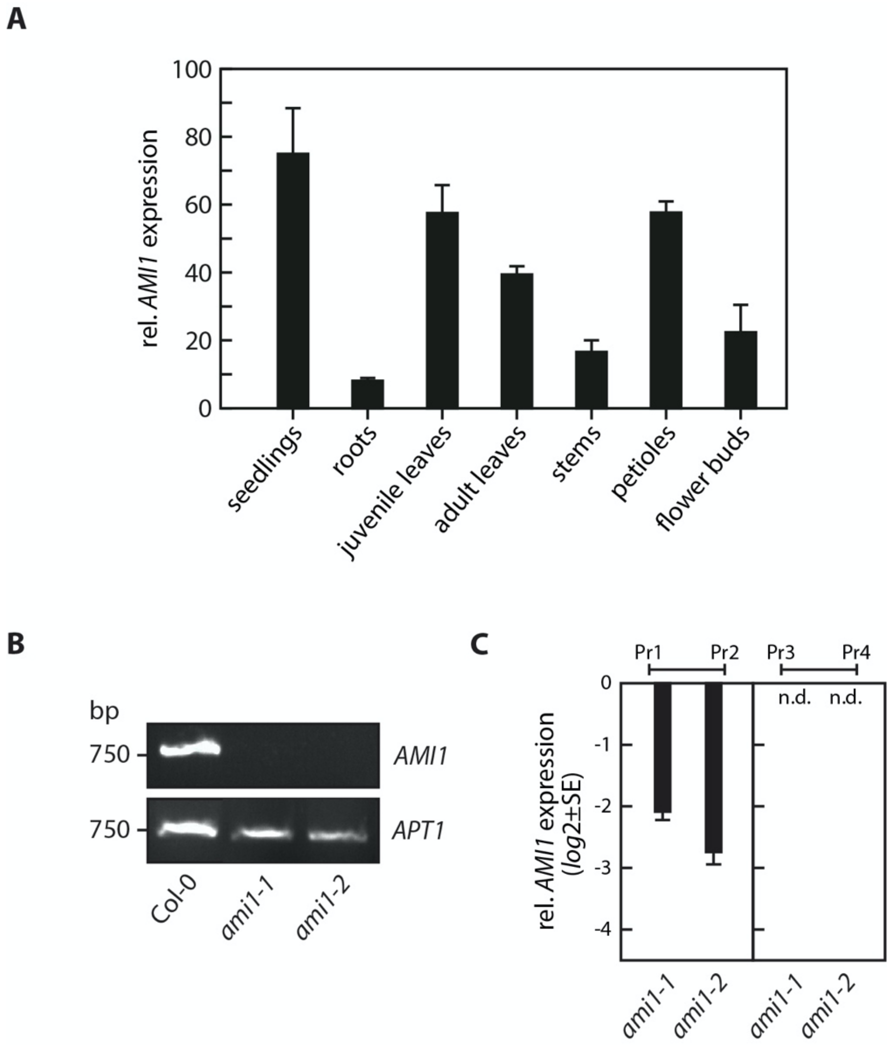

Figure S1. Expression analysis of *AMI1* in wt and genotyping of *ami1* alleles. **(A)** Relative *AMI1* transcript levels in various tissues were measured by qRT-PCR. One hundred milligram plant tissue was harvested for each different sample and used for RNA extraction. Transcript abundance values are given relative to the geometric means of *APT1* and *UBQ10* transcripts. Means are given with their SE (n = 9). **(B)** RT-PCR analysis of *AMI1* gene expression in the T-DNA insertion lines and in wild-type plants. Total RNA from 2-week-old plants was extracted and used for cDNA synthesis. Two gene-specific primers **(Table S1)** amplifying a 750 bp cDNA fragment, spanning the insertion loci, were utilized to investigate *AMI1* transcript levels. Expression of the housekeeping gene *APT1* was used to normalize sample loading. **(C)** Quantitative RT-PCR analysis of *AMI1* expression in the two T-DNA insertion lines relative to *AMI1* gene expression levels in *Arabidopsis* wild type. Gene-specific primers amplifying two different fragments of the *AMI1* gene (5’ and 3’ of the insertion loci, respectively) were chosen **(Table S1).**

Figure S2.

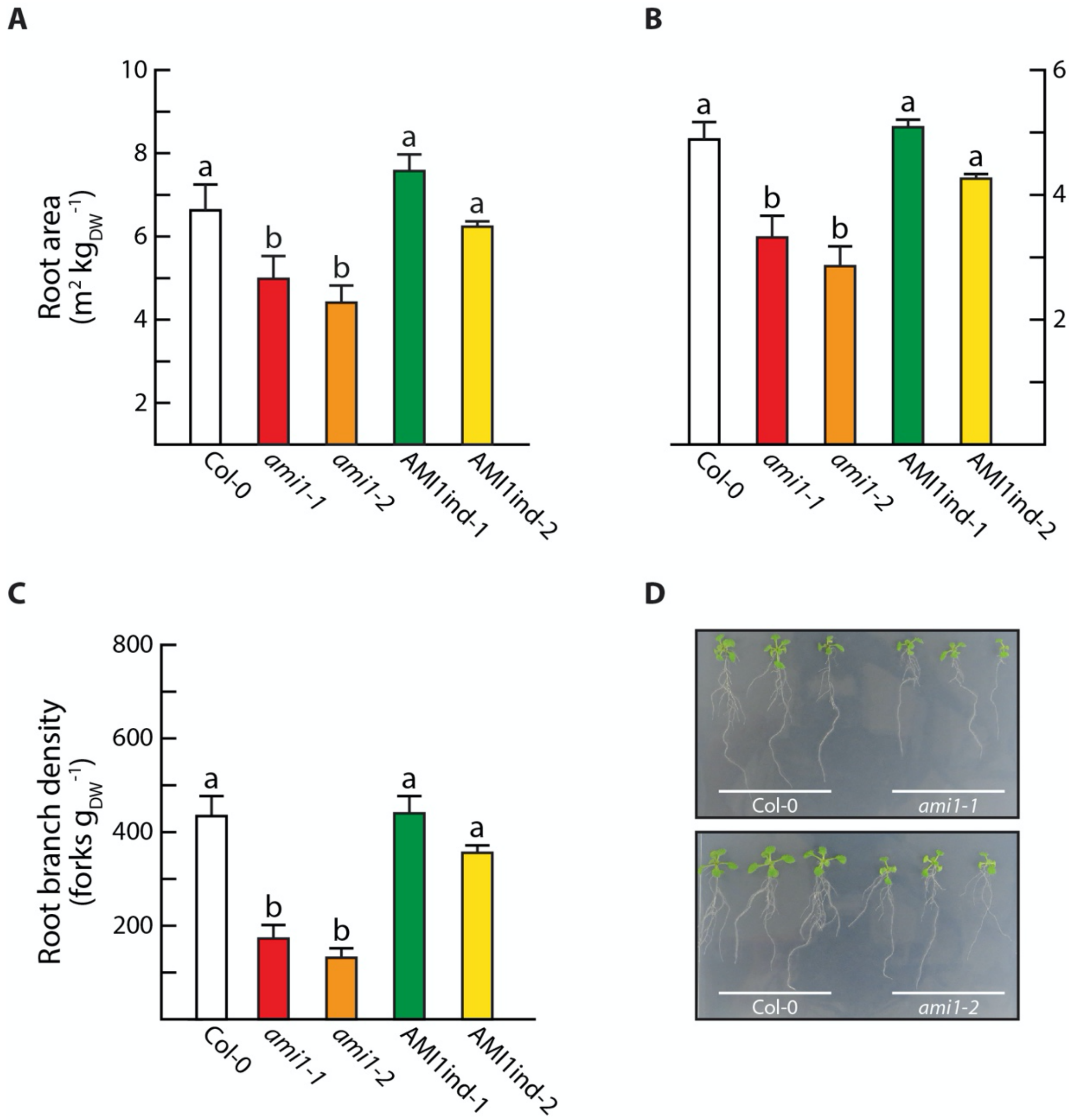
A
B
C
D
Root area
(m² kg_DW^-1)
Root length
(m g_DW^-1)
Root branch density
(forks g_DW^-1)
Col-0
ami1-1
ami1-2
AMI1ind-1
AMI1ind-2
a
b
Col-0
ami1-1
Col-0
ami1-2

Figure S2. Root phenotype of *AMI1* mutant plants. Given are **(A)** the specific root area, **(B)** the specific root length, and **(C)** the specific root branch density of wild-type *Arabidopsis* (Col-0), the two independent *ami1* T-DNA insertion lines, and the two conditional *AMI1* overexression lines grown on ½ MS medium supplemented with 0.5% (w/v) sucrose and vitamins. In order to induce the expression of the transgene in the AMI1ind lines, all Petri dishes contained additionally 10 µM ß-estradiol. The parameters were measured in 12-days-old seedlings. Bars indicate ± SE (n = 23-32). Mean values are significantly (p < 0.05) different among genotypes if superscript letters differ. **(D)** Representative photograph oxf the root phenotype of vertically grown *ami1-1* and *ami1-2* seedlings relative to the wild type (Col-0). Plants were grown on ½ MS medium supplemented with 0.5% (w/v) sucrose and vitamins for 12 days.

Figure S3.

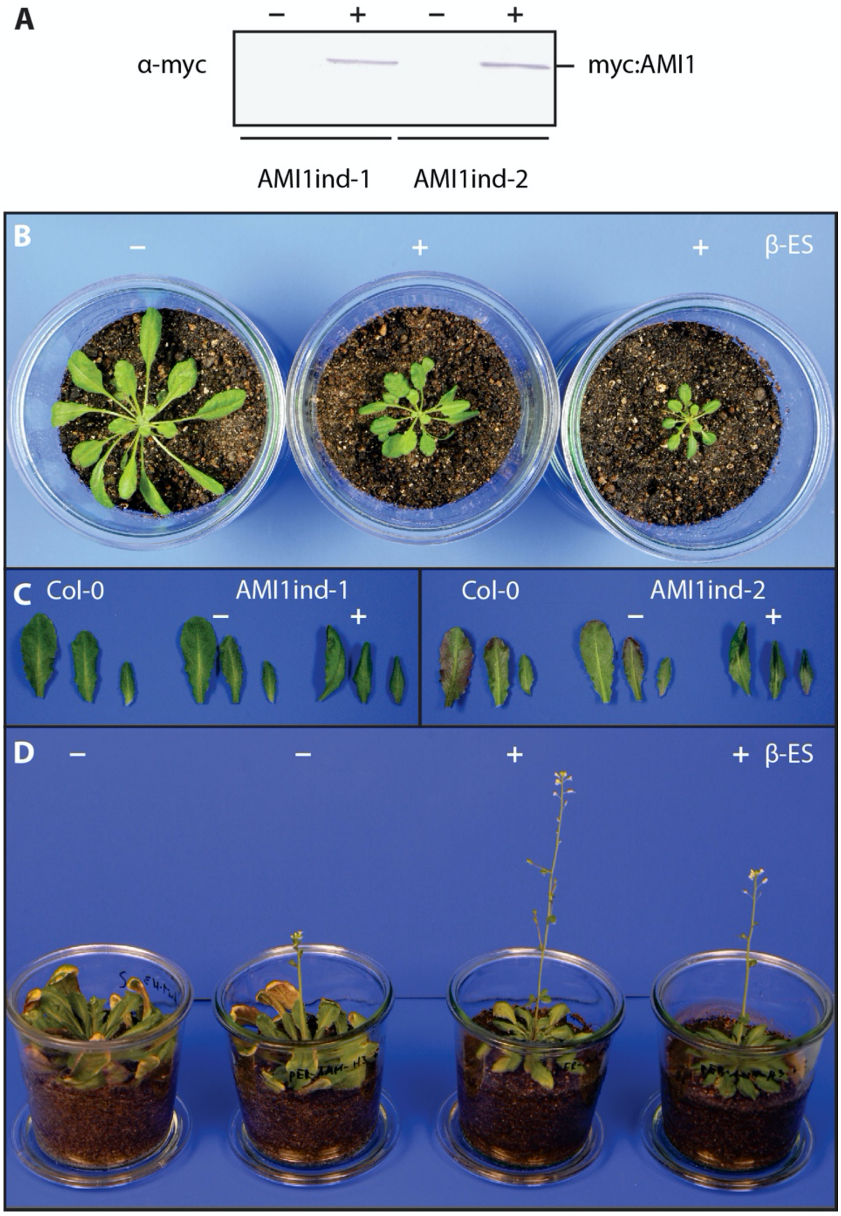
A
–
+
–
+
α-myc
myc:AMI1
AMI1ind-1
AMI1ind-2
B
–
+
+
β-ES
C
Col-0
AMI1ind-1
–
+
Col-0
AMI1ind-2
–
+
D
–
–
+
+ β-ES

Figure S3. Phenotype of conditionally *AMI1* overexpressing AMI1ind lines. **(A)** Detection of recombinant myc:AMI1 protein levels in induced (+) and non-induced (-) AMI1ind lines. 4-week-old plants grown on soil in long days were used for protein isolation followed by immunodetection using a α-myc antibody. **(B)** Images of representative AMI1ind-2 plants non-induced (-) or induced (+) for four weeks after germination. **(C)** Curled leaf phenotype of induced (+) AMI1ind lines in comparison to non-induced (-) transgenic lines and wild-type leaves. **(D)** Phenotype of AMI1ind-2 lines overexpressing AMI1 six weeks after germination. Plants were grown on soil under long day conditions (16 h light). The ectopic expression of myc:AMI1 was achieved by irrigating the plants with 50 µM β-estradiol (β-ES). Non-induced (-) plants, irrigated with water, were taken as a control. Treatment with ß-ES was initiated after the establishment of the first true leaves. It has to be noted that β-ES has no effect on the development of wild-type plants, as wild-type plants were also watered with the hormone.

Figure S4.

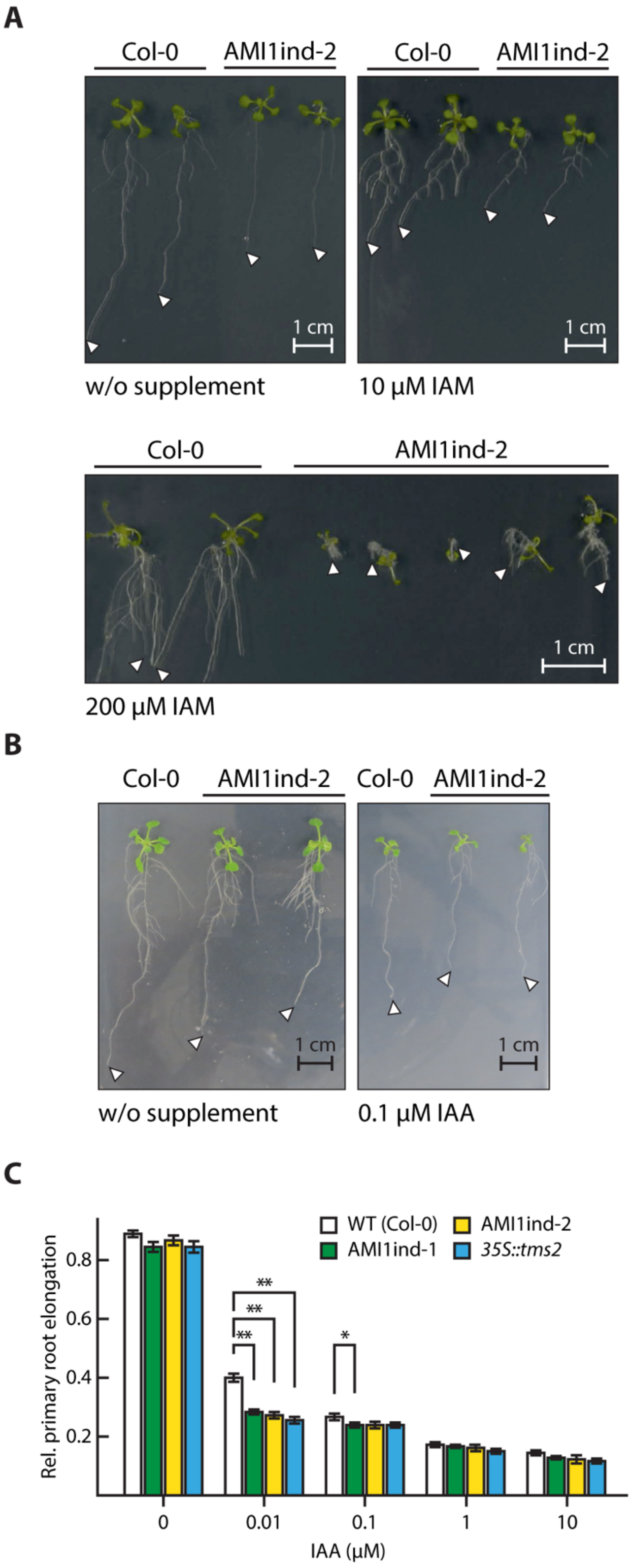
A
Col-0
AMI1ind-2
Col-0
AMI1ind-2
1 cm
1 cm
w/o supplement
10 µM IAM
Col-0
AMI1ind-2
1 cm
200 µM IAM
B
Col-0
AMI1ind-2
Col-0
AMI1ind-2
1 cm
1 cm
w/o supplement
0.1 µM IAA
C
Rel. primary root elongation
0.8
0.6
0.4
0.2
WT (Col-0)
AMI1ind-2
AMI1ind-1
35S::tms2
**
**
**
*
0
0.01
0.1
1
10
IAA (µM)

Figure S4. Root growth responses of conditional *AMI1* overexpression lines towards IAM and IAA in the media. **(A)** Root phenotype of wild-type and AMI1ind-2 seedlings grown without or with IAM in the media. Depicted are 7-day-old seedlings germinated and grown on vertical plates under constant conditions on media containing 10 µM ß-estradiol and either no or 10 µM and 200 µM IAM, respectively. The arrowheads mark the root tips. Similar results were obtained in three independent experiments. **(B)** Representative images showing the phenotype of wild-type and AMI1ind-2 seedlings grown without or with IAA in the media. Depicted are 7-day-old seedlings germinated and grown on vertical plates under constant conditions on media containing 10 µM ß-estradiol and either no or 0.1 µM IAA, respectively. The arrowheads mark the root tips. Similar results were obtained in three independent experiments. **(C)** Comparison of the root growth response towards IAA of two independent AMI1ind lines with wild-type and *35S::tms2* seedlings. Seeds were germinated and grown for 7 days on ½ strength MS plates, before they were transferred onto plates containing indicated amounts of IAA. In order to examine the sensitivity towards IAA in the media without the bias of initially shorter primary roots, the impact of the two compounds was expressed in relative terms. Therefore, the length of the longest primary root of each genotype grown under control conditions was set to a value of 1 and all other roots of the corresponding genotype were expressed relative to this value. The primary root elongation after transfer of seedlings onto media containing indicated concentrations of IAA was quantified. The expression of the transgene was induced by adding 10 µM ß-estradiol to all plates. At least sixteen seedlings of each genotype were measured for each condition. The data represent means ± SE. Asterisks indicate significant differences between the corresponding WT control and the tested genotypes under the described conditions. (Student's *t*-test; *$p \leq 0.05$, **$p \leq 0.01$).

Figure S5.

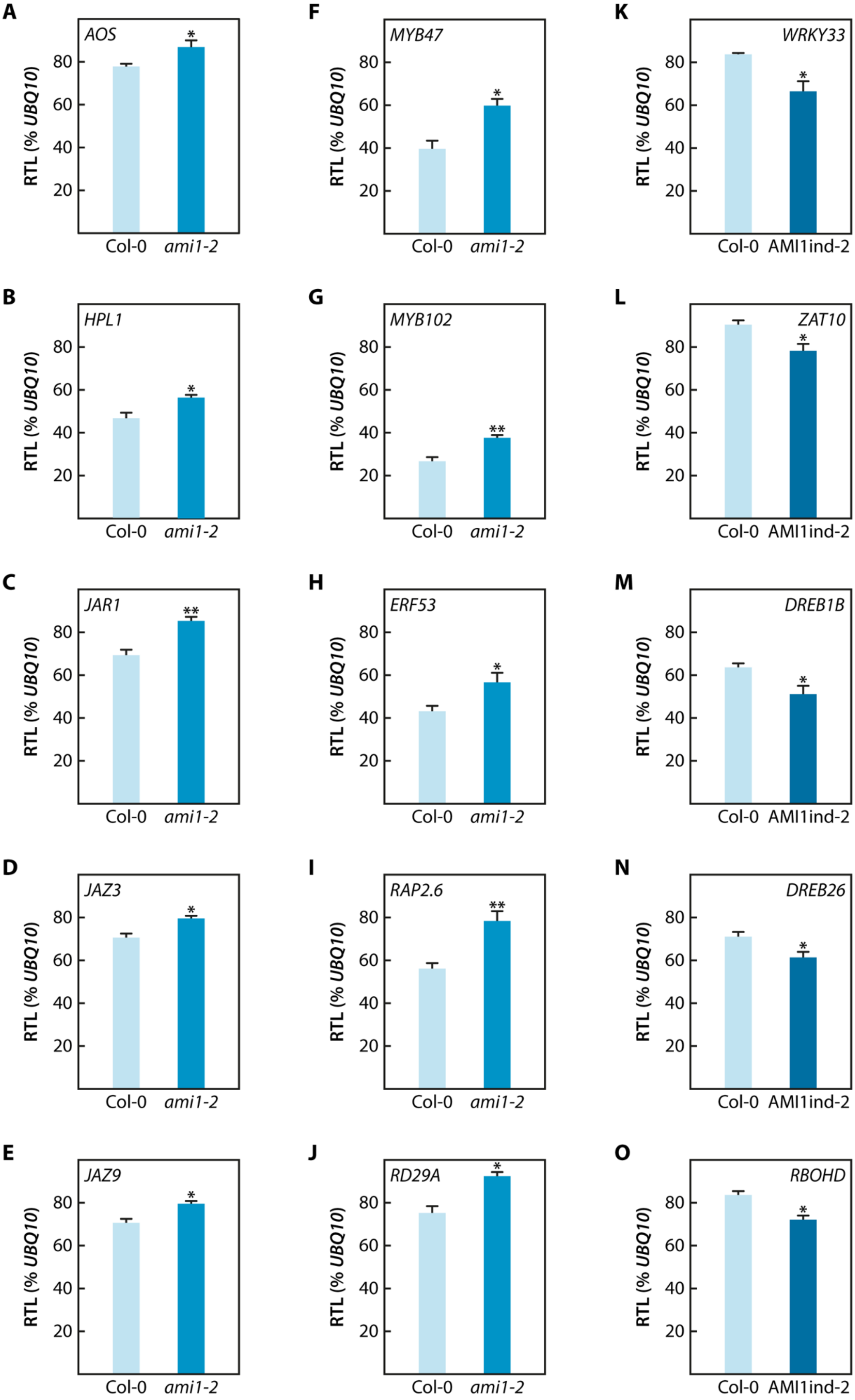

A
AOS
B
HPL1
C
JAR1
D
JAZ3
E
JAZ9
F
MYB47
G
MYB102
H
ERF53
I
RAP2.6
J
RD29A
K
WRKY33
L
ZAT10
M
DREB1B
N
DREB26
O
RBOHD
RTL (% UBQ10)
20
40
60
80
*
**
Col-0
ami1-2
AMI1ind-2

Figure S5. Validation of Microarray Data by qRT-PCR, Related to **Figure 6**. Shown are relative transcript levels (arithmetic mean ± SE, n = 6), normalized to UBQ10 as a constitutively expressed control gene in 12-day-old wt (Col-0), *ami1-2* and AMI1ind-2 seedlings. Asterisks indicate significant differences between the corresponding WT control and the tested genotypes. (Student's *t*-test; *$p \leq 0.05$, **$p \leq 0.01$).

(A) ALLENE OXIDE SYNTHASE (AOS; At5g42650).

(B) HYDROPEROXIDE LYASE 1 (HPL1; At4g15440).

(**C)** JASMONATE RESISTANT 1 (JAR1; At2g46370).

(D) JASMONATE-ZIM-DOMAIN PROTEIN 3 (JAZ3; At3g17860).

(E) JASMONATE-ZIM-DOMAIN PROTEIN 9 (JAZ9; At1g70700).

(F) MYB DOMAIN PROTEIN 74 (MYB47; At4g05100).

(G) MYB DOMAIN PROTEIN 102 (MYB102; At4g21440).

(**H)** ETHYLENE RESPONSIVE FACTOR 53 (ERF53; At2g20880).

(I) RELATED TO AP2 6 (RAP2.6; At1g43160)

(J) RESPONSIVE TO DESICCATION 29A (RD29A; At5g52310).

(K) WRKY DOMAIN PROTEIN 33 (WRKY33; At2g38470).

(L) SALT TOLERANCE ZINC FINGER PROTEIN 10 (ZAT10; At1g27730).

(M) DRE BINDING PROTEIN 1B (DREB1A; At4g25490).

(N) DRE BINDING PROTEIN 26 (DREB26; At1g21910).

(O) RESPIRATORY BURST OXIDASE HOMOLOGUE D (RBOHD; At5g47910).

Data are from an experiment independent of the data presented in **Table S2**.

Figure S6.

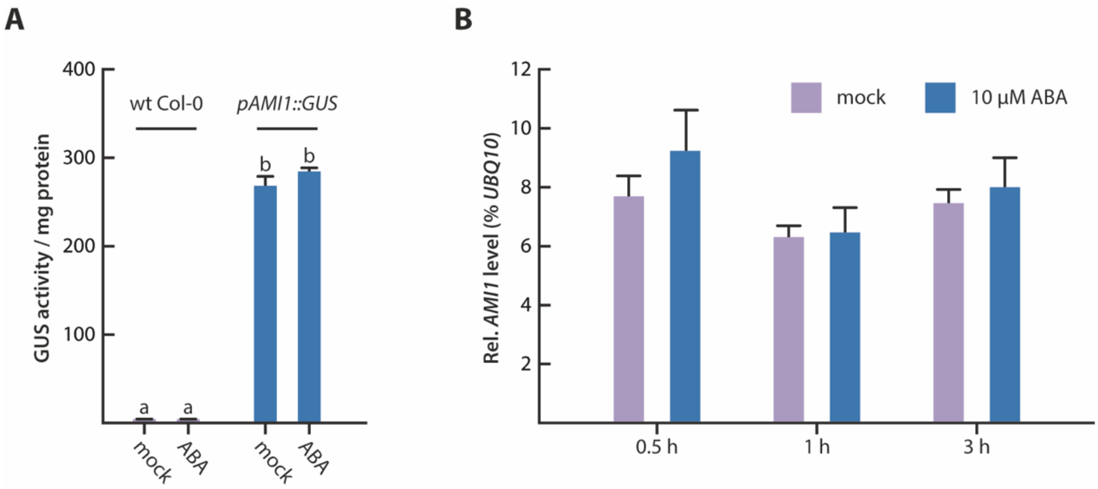

Figure S6. Effect of ABA on *AMI1* gene expression. **(A)** Fluorometric quantification of GUS activity in *pAMI1::GUS* seedlings that were either mock (MeOH) treated or incubated with 10 µM ABA for 2 h. Lowercase letters indicate statistically different groups ($p \leq 0.001$). **(B)** Shown are relative transcript levels for *AMI1* (arithmetic mean ± SE, n = 6), normalized to UBQ10 as a constitutively expressed control gene in 12-day-old wt plants treated for 0.5 h, 1 h and 3 h, respectively, with 10 µM ABA.
